# Absence of cytoglobin promotes multiple organ abnormalities in aged mice

**DOI:** 10.1038/srep24990

**Published:** 2016-05-05

**Authors:** Le Thi Thanh Thuy, Tuong Thi Van Thuy, Yoshinari Matsumoto, Hoang Hai, Yoshihiro Ikura, Katsutoshi Yoshizato, Norifumi Kawada

**Affiliations:** 1 Department of Hepatology, Graduate School of Medicine, Osaka City University, Osaka, Japan; 2 Department of Medical Nutrition, Graduate School of Human Life Science, Osaka City University, Osaka, Japan; 3 Department of Pathology, Takatsuki General Hospital, Takatsuki, Osaka, Japan; 4 PhoenixBio Co. Ltd., Hiroshima, Japan

## Abstract

Cytoglobin (Cygb) was identified in hepatic stellate cells (HSCs) and pericytes of all organs; however, the effects of Cygb on cellular functions remain unclear. Here, we report spontaneous and age-dependent malformations in multiple organs of *Cygb*^−/−^ mice. Twenty-six percent of young *Cygb*^−/−^ mice (<1 year old) showed heart hypertrophy, cystic disease in the kidney or ovary, loss of balance, liver fibrosis and lymphoma. Furthermore, 71.3% (82/115) of aged *Cygb*^−/−^ mice (1–2 years old) exhibited abnormalities, such as heart hypertrophy and cancer development in multiple organs; by contrast, 5.8% (4/68) of aged wild-type (WT) mice had abnormalities (*p* < 0.0001). Interestingly, serum and urine analysis demonstrated that the concentration of nitric oxide metabolites increased significantly in *Cygb*^−/−^ mice, resulting in an imbalance in the oxidative stress and antioxidant defence system that was reversed by N^G^-monomethyl-L-arginine treatment. A senescent phenotype and evidence of DNA damage were found in primary HSCs and the liver of aged *Cygb*^−/−^ mice. Moreover, compared with HSC^+/+^, HSC^−/−^ showed high expression of Il-6 and chemokine mRNA when cocultured with mouse Hepa 1–6 cells. Thus, the absence of Cygb in pericytes provokes organ abnormalities, possibly via derangement of the nitric oxide and antioxidant defence system and through accelerated cellular senescence.

Cytoglobin (Cygb) was originally identified in 2001 as a protein upregulated in activated rat hepatic stellate cells (HSCs) under pro-fibrotic conditions; accordingly, Cygb was originally termed stellate cell activation-associated protein (STAP)^1^. Cygb is the fourth globin identified in mammals^2^,^3^, with human Cygb displaying ~25% amino acid identity with vertebrate myoglobin (Mb) and haemoglobin (Hb) and 16% identity with human neuroglobin (Ngb). Small gas molecules, such as oxygen (O_2_), carbon monoxide (CO) and nitric oxide (NO), bind reversibly to the haem iron of Cygb in a manner similar to that of the other globins. Mb shows tissue-restricted distribution in cardiomyocytes and skeletal myofibres, Hb in erythrocytes, and Ngb in the nervous system. In contrast, Cygb is expressed ubiquitously in the cytoplasm of pericytes in many organs, including the brain, thymus, heart, lung, liver, kidney, small intestine and spleen^4^. An interesting aspect of Cygb expression is its presence in visceral cells with the ability to store vitamin A. In normal and fibrotic human livers, Cygb was expressed in HSCs but not in hepatocytes, thereby serving as a marker of quiescent HSCs^5^.

Cygb functions include (1) O_2_ storage, diffusion and sensing for cellular respiration and metabolism; (2) NO scavenging; and (3) involvement in hypoxia and oxidative stress. First, Cygb exhibits intrinsic O_2_-binding capacity; its haem iron demonstrates similar affinities for exogenous ligands and equilibrium constants for O_2_ as those observed for Mb^1^,^3^. The distribution of Cygb in fibroblast-like cells suggests that it functions as an O_2_ sensor involved in cell proliferation and, possibly, O_2_ diffusion for collagen synthesis during wound healing^6^, although such cells are not generally associated with high metabolic rates and O_2_ consumption.

Second, Cygb displays nitric oxide dioxygenase (NOD) activity^7^,^8^. Smagghe and colleagues examined the NOD activity of various globins in their oxy-ferrous state and showed that human Ngb and Cygb, rice nsHb (riceHb1), *Synechocystis* Hb (cyanoglobin, SynHb), and horse heart Mb rapidly destroy NO *in vitro*; among these, Cygb showed the highest consumption rate^9^. At low O_2_ levels (0–50 mM), Cygb and other cellular reductants regulated the rate of NO consumption in response to O_2_ concentration changes, showing ~500-fold greater sensitivity to changes in O_2_ level than Mb^10^. The NO-scavenging function of Cygb was found to protect the NO-sensitive aconitase, decrease peroxynitrite (ONOO^−^) formation and protect cellular respiration^8^. The Cygb expression patterns in human and rat hippocampus showed co-expression and subsequent upregulation of Cygb and neuronal NO synthase (nNOS) following chronic restraint stress^11^. The high level of Cygb and nNOS co-expression supports the hypothesised involvement of Cygb in NO metabolism. Accumulation of ONOO^−^ and other nitrosative molecules results in nitrosative stress, which might affect protein tyrosine residues, metalloproteins, lipids and nucleic acids^12^,^13^. Thus, the NO-scavenging function of Cygb seems to be crucial for protecting cells/tissues from NO accumulation.

Finally, the hypoxia and oxidative responses of Cygb have been examined in various tumour cell lines, including sporadic head-and-neck squamous cell carcinoma^14^ and human glioblastoma multiform^15^, as well as in animal models, such as models of murine embryogenesis and processes in adult tissues^16^. Furthermore, *in vitro* and *in vivo* overexpression of Cygb in rat HSCs protected these cells against oxidative stress and inhibited their differentiation into an activated phenotype^17^. Recently, Latina *et al*. reported that *Cygb* is transcriptionally regulated by ΔNp63 in primary epithelial cells (keratinocytes) and in cancer cells (H226, MCF-7) under normal proliferating conditions (normoxia) and following oxidative stress^18^. These reports suggest that in addition to functioning as a gas carrier, Cygb might act as a cytoprotective factor under conditions of hypoxia and oxidative stress.

To study the biological function of Cygb at the tissue level, we generated *Cygb*-deficient (*Cygb*^−/−^) mice and reported their high susceptibility to tumour development in the liver and lungs when treated with *N*, *N*-diethylnitrosamine (DEN)^19^. Furthermore, *Cygb*^−/−^ mice exhibited increases in liver inflammation, fibrosis and cancer development in a non-alcoholic steatohepatitis (NASH) model induced by a choline-deficient L-amino acid-defined diet via activation of the oxidative stress pathway^20^. Therefore, the absence of Cygb probably promotes fibrotic and carcinogenic processes in chronic liver diseases. However, it remains unclear whether CYGB plays a protective role in various organs under physiological conditions.

During the maintenance and propagation of our *Cygb*^−/−^ mice, we detected the formation of age-dependent abnormalities in these mice. The main abnormalities in the *Cygb*^−/−^ mice under 1 year of age (hereafter, called young mice) were heart hypertrophy and cystic diseases in the kidney and ovary, and the less frequent abnormalities included paralysis, loss of balance, liver fibrosis and lymphoma. In contrast, a total of 82 out of 115 (71.3%) *Cygb*^−/−^ mice ranging from 1–2 years of age (hereafter, referred to as aged mice) displayed multiple organ abnormalities, including heart hypertrophy and tumours in the lung, liver, ovary, small intestine and lymphatic organs. Interestingly, the concentration of NO metabolites was increased significantly in the serum and urine of *Cygb*^−/−^ mice compared with that in WT counterparts. These data confirmed an imbalance in the oxidative stress and antioxidant defence system with increased expression of oxidative stress-related genes, in contrast to the downregulation of antioxidative genes. Thus, the presence of Cygb in the pericytes of all organs serves an important function in maintaining homeostasis of the antioxidant system.

## Results

### Multi-organ abnormalities in Cygb deficient mice

**Figure 1:**
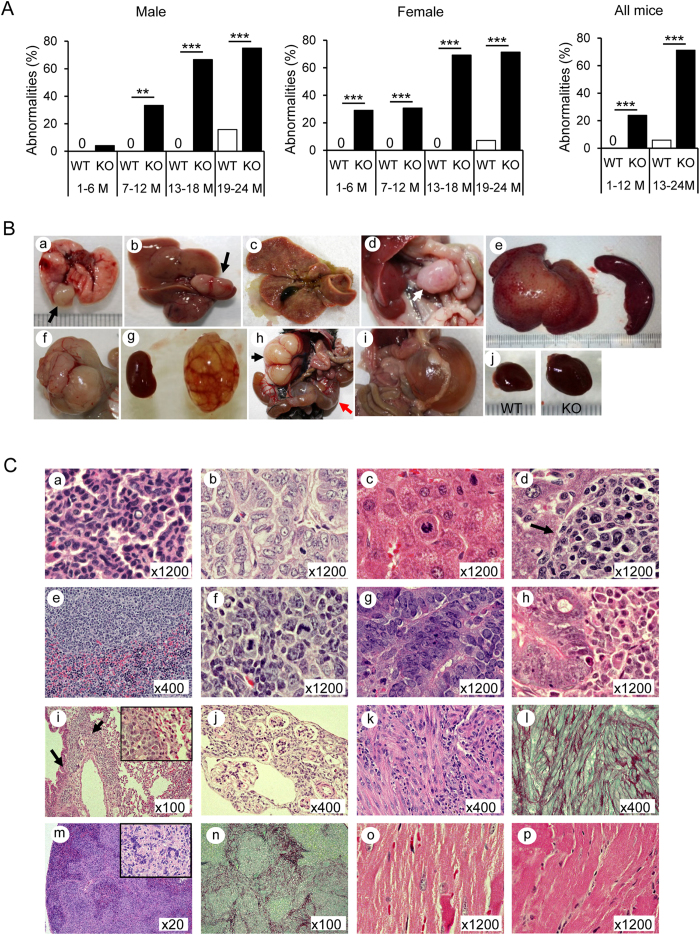
Multiple organ abnormalities in *Cygb*^−/−^ mice. (**A**) Percentage of abnormalities found in male (left panel), female (middle panel), and all (right panel) wild-type (WT) and *Cygb*^−/−^ (KO, knockout) mice in four age groups: 1–6 months of age (1–6 M), 7–12 months (7–12 M), 13–18 months (13–18 M), and 19–24 months (19–24 M). Open bar, WT; closed bar, KO. Data represent the mean ± SD; n = 15–56 per group; **p < 0.01; ***p < 0.001. (**B**) Macroscopic findings in *Cygb*^−/−^ mice: tumour nodule (black arrow) of the lung at 18 M (a); liver tumour, 17 M (b); liver cholestasis, 5 M (c); swelling of mesenteric lymph node, 23 M (d, white arrow); hepatosplenomegaly, 11 M (e); intestinal tumour, 24 M (f); kidney cyst, 4 M (g); kidney deformity (black arrow) and cyst of uterus (red arrow), 6 M; mesenteric cyst, 10 M (i); heart hypertrophy, 22 M (j). (**C**) Representative haematoxylin and eosin (H&E)-stained sections of *Cygb*^−/−^ mice with adenoma (a) and adenocarcinoma (b) of the lung; hepatocellular carcinoma (HCC) (c); lymphoma in the liver (d, arrow), spleen (e), and mesenteric lymph node (f); intestinal adenoma (g); intestine lymphoma (h), which was metastatic to the lung (i, arrows; right inset, ×1200); kidney cysts (j); H&E and Sirius Red and Fast Green (SiR-FG) staining of a renal myomatous lesion (k, l) and spleen fibrosis (m, n; right inset, ×800); hypertrophy of cardiomyocytes in KO mice compared with WT mice (o, WT; p, KO).

Previously, we generated Cygb-deficient mice by deleting exon 1 of the mouse *Cygb* gene and backcrossing on the C57BL/6J background^19^. The mice that were homozygous for the disrupted allele appeared normal both morphologically and histopathologically 1 month (M) after birth. However, we found a time-dependent emergence of abnormalities in various organs of *Cygb*^−/−^ mice. Among 92 *Cygb*^−/−^ young mice of both sexes, 24 (26.0%) showed abnormalities. Nine mice had heart hypertrophy at 10 M, five mice had kidney cysts at 4 M, five had liver fibrosis and lymphoma from 5–11 M, two displayed loss of balance at 2 M, one mouse each had a cyst in the uterus or ovary at 4 M, and one displayed paralysis of the rear legs at 4 M. Meanwhile, none of the 135 WT young mice showed any abnormalities (*p* < 0.0001 by the Fisher exact test, two-tailed) (Fig. 1A). Importantly, aged *Cygb*^−/−^ mice displayed a significantly greater number of abnormalities (82/115; 71.3%) compared with the number observed in aged WT mice (4/68; 5.8%) (*p* < 0.0001), and some *Cygb*^−/−^ mice showed multiple organ abnormalities (Fig. 1A and Table 1). The macroscopic abnormalities in *Cygb*^−/−^ mice included lung tumour (Fig. 1Ba), liver tumour (Fig. 1Bb), liver cholestasis (Fig. 1Bc), swelling of the mesenteric lymph node (Fig. 1Bd), hepatosplenomegaly (Fig. 1Be), intestinal tumour (Fig. 1Bf), kidney cyst (Fig. 1Bg), kidney deformity and uterine cyst (Fig. 1Bh), mesenteric cyst (Fig. 1Bi), and heart hypertrophy (Fig. 1Bj). Histopathological analysis of lungs from *Cygb*^−/−^ mice at 18 M revealed that the primary tumour types were adenoma (Fig. 1Ca) and adenocarcinoma (Fig. 1Cb). The livers of *Cygb*^−/−^ mice at 17 M exhibited hepatocellular carcinoma (HCC) (Fig. 1Cc). Systemic lymphoma in *Cygb*^−/−^ mice at 11 M occurred in the liver (Fig. 1Cd), spleen (Fig. 1Ce) and mesenteric lymph node (Fig. 1Cf). All lymphoma cases were immunohistochemically stained for CD3 and CD22, markers of the T and B cells, respectively. We found that these lymphomas were derived from T cells but not B cells (see Supplementary Fig. S2). Intestinal adenoma (Fig. 1Cg) was found at 21 M. Intestinal lymphoma (Fig. 1Ch), which was metastatic to the lung (Fig. 1Ci), was found at 24 M. A cyst in the kidney (Fig. 1Cj) was found at 4 M. A potential renal myomatous lesion (Fig. 1Ck) was accompanied by robust collagen fibres, as shown by Sirius red and fast green (SiR-FG) staining (Fig. 1Cl). Fibrosis of the spleen was demonstrated by haematoxylin and eosin (H&E; Fig. 1Cm) and SiR-FG (Fig. 1Cn) staining. Cardiomyocyte hypertrophy (Fig. 1Co, WT; Fig. 1Cp, KO) was observed. Heart hypertrophy was further demonstrated by the increase in the heart weight (HW)/body weight (BW) ratio and by the increase in the heart size in terms of length and width (Supplementary Fig. S1A) compared with WT. The HW/BW ratio in young *Cygb*^−/−^ mice was 16.9% greater than that in WT. However, the HW/BW ratio in aged *Cygb*^*−/−*^ mice showed a 41.2% increase (*p* < 0.0001) compared with WT (Supplementary Fig. S1A). The length of the heart increased significantly in young *Cygb*^−/−^ mice, and both the length and width increased significantly in aged mice compared with those in WT mice (Supplementary Fig. S1B). Microscopic analysis demonstrated enlarged hearts (Supplementary Fig. S1C) in Cygb^−/−^ mice compared with those in WT. Interstitial fibrosis was observed in *Cygb*^−/−^ mice, as indicated by SiR-FG staining (Supplementary Fig. S1D).

### Liver abnormalities in Cygb-deficient mice

**Figure 2:**
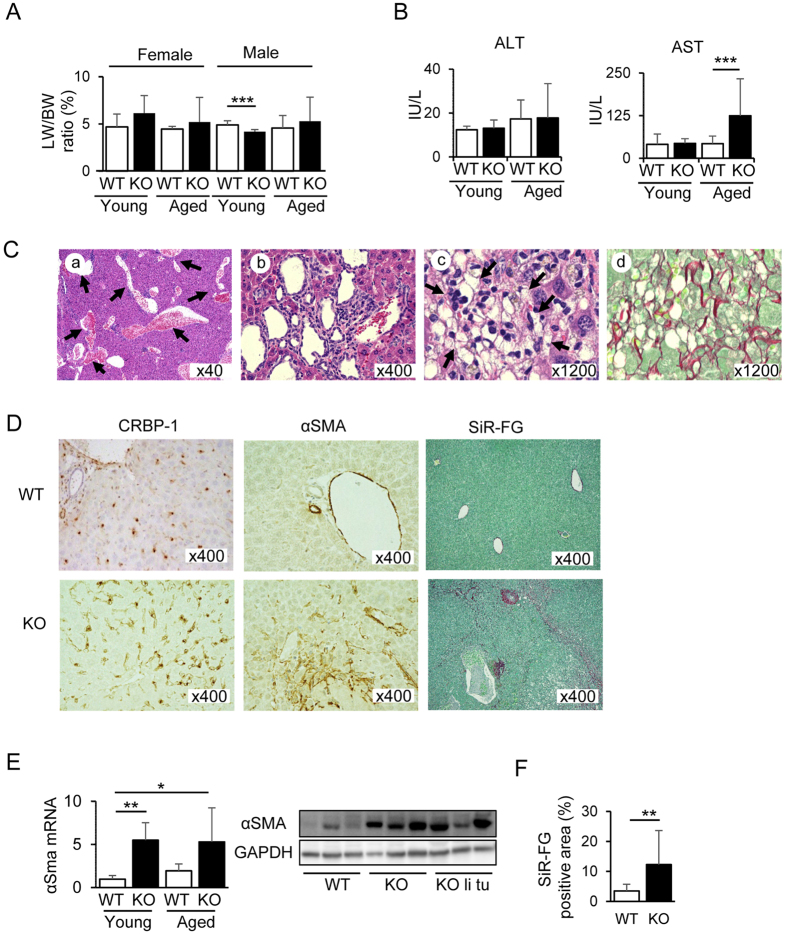
Liver fibrosis in *Cygb*^−/−^ mice. (**A**) Liver weight (LW): body weight (BW) ratio of male and female mice. (**B**) Alanine aminotransferase (ALT) and aspartate aminotransferase (AST) levels in the serum. (**C**) H&E staining of liver sections from aged KO mice showing dilation of the portal vasculature (a; arrow) hyperplasia of the bile duct (b) and hepatic stellate cells (HSC) (c; arrow) SiR-FG staining of HSC hyperplasia showing severe accumulation of collagen fibres (d). (**D**) Representative liver sections from aged WT and KO mice stained for cellular retinol binding protein 1 (CRBP-1) (left-panel) and α smooth muscle actin (αSMA) (middle-panel) or stained with SiR-FG (right-panel). (**E**) Quantitative real-time PCR analysis of αSMA expression at the mRNA level. Right inset, western blotting analysis for αSMA from homogenised liver tissues of aged WT, KO, and KO liver tumours (KO li tu). GAPDH was used as a loading control. All gels were run under the same experimental conditions. Cropped gels were used, and full-length gels are presented in Supplementary Fig. S3. (**F**) The Sirius red-positive area was quantified in aged WT and KO liver. Open bar, WT; closed bar, KO. Young mice: ≤12 months of age; aged mice: 13–24 months. Data represent the mean ± SD; n = 15–35 per group. *p < 0.05; **p < 0.01; ***p < 0.001.

After discovering Cygb in the HSCs of rat liver, we focused our efforts on examining the liver of *Cygb*^−/−^ mice. We observed spontaneous development of liver tumours in 22.6% of aged *Cygb*^−/−^ mice (Table 1). The liver weight (LW)/BW ratio tended to increase in all female and aged male mice (Fig. 2A). Although the alanine aminotransferase (ALT) level was similar between *Cygb*^−/−^ and WT mice, the aspartate aminotransferase (AST) level was significantly increased in aged *Cygb*^−/−^ mice compared with WT controls (Fig. 2B). In addition, we found other liver abnormalities, such as dilation of the portal vasculature (Fig. 2Ca), and hyperplasia of the bile duct (Fig. 2Cb) and HSCs (Fig. 2Cc,d). In accordance with these observations, SiR-FG staining and quantification revealed fibrosis in the liver of aged *Cygb*^−/−^ mice, but not WT, in the absence of stimulants (Fig. 2D,F). Cellular retinol binding protein-1 (CRBP-1) immunostaining indicated the presence of HSCs in the absence of Cygb (Fig. 2D), similar to findings for the WT mouse liver. In addition, the expression of α smooth muscle actin (αSMA), a marker of activated HSCs, was increased at both the mRNA and protein levels in *Cygb*^−/−^ mice (Fig. 2D,E). These data suggest that Cygb deficiency induced mild hepatocyte injury, activated HSCs, and stimulated the development of spontaneous liver fibrosis in an age-dependent manner.

### Possible involvement of NO and oxidative stress in the liver damage of Cygb-deficient mice

**Figure 3:**
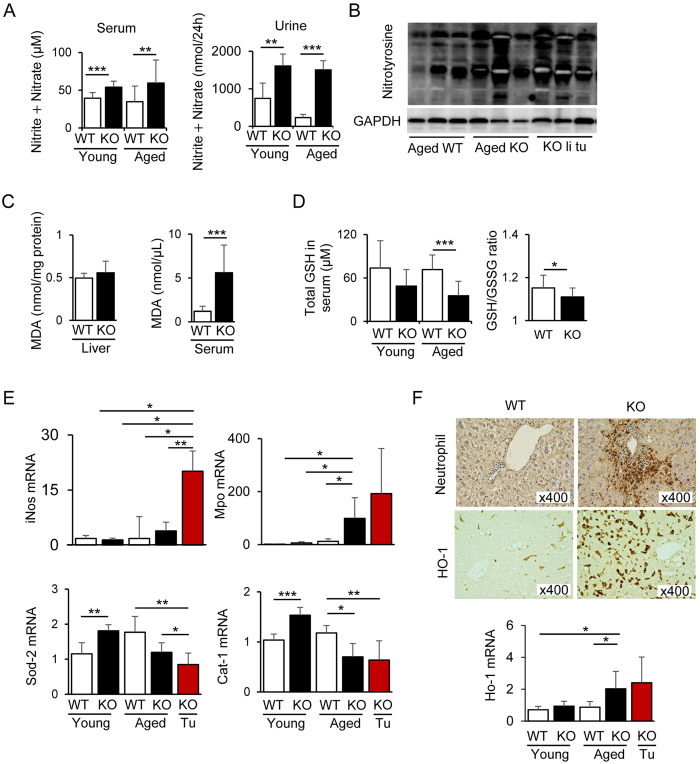
Imbalance of oxidative stress and the antioxidant system in *Cygb*^−/−^ mice. (**A**) Total concentrations of nitrite and nitrate, oxidised forms of nitrogen, in the serum (left-panel) and urine (right-panel) of young and aged WT and KO mice. (**B**) Immunoblots for nitrotyrosine (NT), an indicator or marker of cell damage, inflammation and NO (nitric oxide) production, in aged WT, KO non-tumour, and KO liver tumour area. GAPDH, loading control. All gels were run under the same experimental conditions. Cropped gels were used, and full-length gels are presented in Supplementary Fig. S4. (**C**) Concentration of malondialdehyde (MDA), an end product of lipid peroxidation, in the liver and serum of young WT and KO mice. (**D**) Total glutathione (GSH) in the serum of young and aged WT and KO mice (left-panel) and GSH/oxidised GSH (GSSG) ratio in the liver tissues (right-panel) of aged WT and KO mice. (**E**) The increased hepatic mRNA transcript levels of the pro-oxidant genes inducible nitric oxide synthase (iNos), myeloperoxidase (Mpo) (top panels) are opposite to the decreased expression of the antioxidant genes superoxide dismutase 2 (Sod-2) and catalase-1 (Cat-1) (bottom panels). (**F**) Liver sections of aged WT and KO mice were immunohistochemical stained for neutrophil and haem oxygenase-1 (HO-1). Bottom inset, hepatic mRNA levels of Ho-1. Open bar, WT; closed bar, KO. Young mice: ≤12 months of age; aged mice: 13–24 months. Data represent the mean ± SD; n = 10–15 per group. *p < 0.05; **p < 0.01; ***p < 0.001.

Cygb scavenges NO and other reactive oxygen species (ROS)^21^. Therefore, we hypothesised that the liver and the other organs might suffer from high concentrations of NO and ROS in the absence of Cygb. As shown in Fig. 3A (left), the concentration of nitrite + nitrate, oxidised forms of nitrogen, in the serum of *Cygb*^−/−^ mice was increased significantly compared with that in WT mice, and this difference was observed in both young and aged mice. In addition, the nitrite + nitrate concentration in the urine of *Cygb*^−/−^ mice was elevated markedly compared with that in WT mice (Fig. 3A, right). These results indicated that the absence of Cygb augments production of NO in the whole body. In addition, we detected robust expression of nitrotyrosine (NT) protein adducts in the liver and liver tumour lesions in aged *Cygb*^−/−^ mice compared with WT (Fig. 3B), implying an enhanced reaction of NO with superoxide anion (O_2_^−^) to produce ONOO^−^. It is plausible that long term increases in NO production in the body would induce vasodilation and increased cardiac volume load, which might explain the heart hypertrophy observed in *Cygb*^−/−^ mice^22^.

With regard to ROS productions, we assessed the level of malondialdehyde (MDA), an end product of lipid peroxidation, in the liver and serum of young mice. As shown in Fig. 3C, MDA changed slightly in the liver but increased significantly in the serum of young *Cygb*^−/−^ mice compared with WT, indicating that ROS production was augmented in the absence of Cygb.

Extracellular NO reacts and consumes intracellular glutathione^23^, and it also triggers cellular oxidative stress^24^. Therefore, we measured the glutathione (GSH) concentration in the serum and liver. As expected, the total GSH in the serum showed a decreasing tendency in young *Cygb*^−/−^ mice and a significant decrease in aged *Cygb*^−/−^ mice compared with WT mice (Fig. 3D, left). The redox status expressed as the GSH:oxidised GSH (GSSG) ratio was lower in the liver tissues from aged *Cygb*^−/−^ mice compared with WT, suggesting enhanced oxidative stress in *Cygb*^−/−^ mice (Fig. 3D, right).

We examined the expression of 84 key genes involved in the oxidative stress and antioxidant defence system using a PCR array in *Cygb*^−/−^ and WT mouse livers. Table 2 shows the most downregulated or upregulated genes of this array. Livers from 1-month-old *Cygb*^−/−^ mice showed downregulation of almost all antioxidative genes, including glutathione peroxidase 3 (Gpx3), flavin-containing monooxygenase 2 (Fmo-2), and serine (or cysteine) peptidase inhibitor (Serpinb1b), compared with WT. Such downregulation of antioxidative genes was more prominent in 14-month-old *Cygb*^−/−^ mice and was accompanied by increased expression of pro-oxidant genes, such as myeloperoxidase (Mpo), inducible NO synthase 2 (iNos), nucleoredoxin, and eosinophil peroxidase. Quantitative real-time (qRT)-PCR analysis further confirmed the significant increase in the mRNA expression of iNos and Mpo and the downregulation of the superoxide dismutase 2 (Sod-2) and catalase-1 (Cat-1) mRNA expression in aged *Cygb*^−/−^ mice compared with WT, particularly in the tumour lesions (Fig. 3E). Consistent with the increased mRNA transcript level of Mpo, immunohistochemical staining showed the robust accumulation of neutrophils in the liver of *Cygb*^−/−^ mice (Fig. 3F). Thus, the absence of Cygb induced an imbalance between ROS production and the endogenous antioxidant system.

Haem oxygenase-1 (HO-1), also known as heat shock protein 32 (HSP32)^25^, is another component of the cellular defence mechanism against oxidative stress. Here, we detected increased HO-1 expression at the protein and mRNA levels in the livers of aged *Cygb*^−/−^ mice and in tumour lesions (Fig. 3F). The results obtained from this liver analysis implied that the loss of Cygb, which is dominantly expressed in the pericytes of all organs, induces oxidative stress conditions in the whole body, which consequently promote multiple organ abnormalities.

### Premature senescence of HSCs in Cygb^−/−^ mice

**Figure 4:**
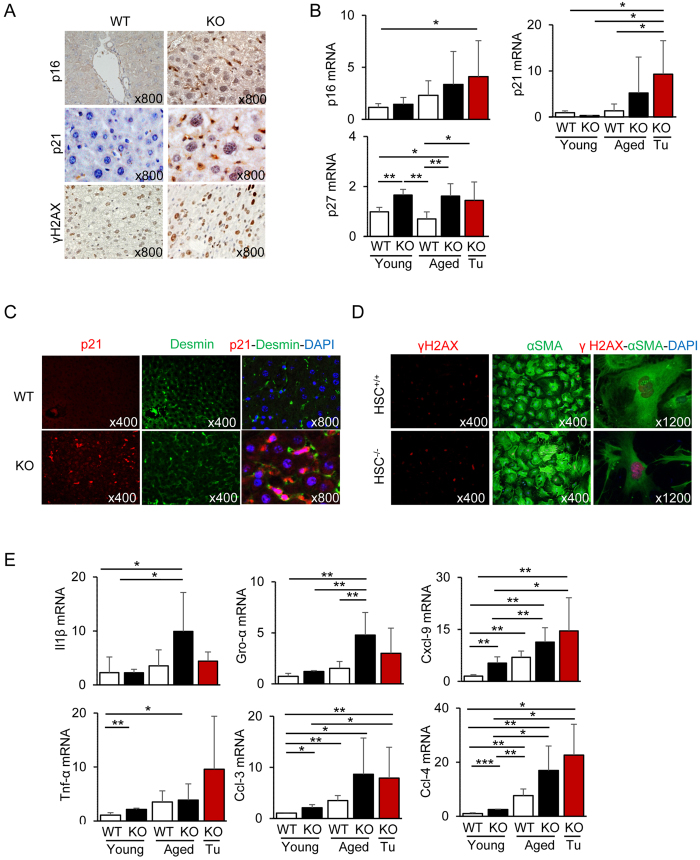
Senescence phenotype in aged *Cygb*^*−/−*^ mice. (**A**) Liver sections of aged WT and KO mice were immunostained for p16, p21, and phosphorylated γH2AX (pSer139). Note that the p16- and p21-positive cells were sinusoidal cells but not hepatocytes. (**B**) Hepatic mRNA levels of p16, p21 and p27. (**C**) Liver sections of aged WT and KO mice were double-stained for p21 (red) and desmin (green) immunofluorescence. Nuclei were stained with 4′,6-diamidino-2-phenylindole (DAPI) (blue). (**D**) Primary young HSC^+/+^ and HSC^−/−^ at day 7 were used for immunofluorescence staining for γH2AX (pSer139). Hepatic mRNA levels of interleukin (Il) 1β, growth regulated alpha (Gro-α), C-X-C motif chemokine (Cxcl)-9, tumour necrosis factor (Tnf)-α, C-C motif chemokine (Ccl)-3, and Ccl-4 (**E**). Open bar, WT; Closed bar, KO; Data represent the mean ± SD; n = 10–15 per group. *p < 0.05; **p < 0.01; ***p < 0.001.

Various cellular stresses, such as oncogene activation, oxidative stress and DNA damage, can induce cellular senescence^26^. The senescence-associated secretory phenotype (SASP), which includes various inflammatory and tumour-promoting factors in HSCs, has crucial roles in promoting obesity-associated HCC development in mice^27^. Therefore, we examined whether the loss of Cygb induces HSC senescence in our model. We detected cells positive for p16 and p21 (two senescence-related genes or senescence inducers) in the sinusoidal cells but not the hepatocytes of aged *Cygb*^−/−^ mice. WT mouse liver showed negligible expression of these proteins (Fig. 4A). Consistent with these results, qRT-PCR analysis showed elevated mRNA expression of p16, p21 and p27 in the liver and liver tumours of aged *Cygb*^−/−^ mice compared with WT controls (Fig. 4B). Double immunofluorescence staining of p21 and desmin, a marker of HSCs, showed localization of p21 in the nucleus and desmin in the cytoplasm of HSCs (Fig. 4C). In addition, positive staining was observed for a marker of oxidative stress induced-DNA double strand breaks, phosphorylated γH2AX (pSer139), in a non-tumourous area of both aged WT and *Cygb*^−/−^ mice, but the level was markedly elevated in Cygb-deficient mice (Fig. 4A). In a 400× field, 42.3 ± 10.9 cells were positive for phosphorylated γH2AX in the liver of aged *Cygb*^−/−^ mice, which differed significantly from the 15.3 ± 4.57 positive cells observed in the WT counterparts (*p* = 0.0037 by a two-tailed *t*-test). We further examined the expression of phosphorylated γH2AX in HSCs isolated from 12-week-old WT and *Cygb*^−/−^ mice (Fig. 4D). A significantly greater number of phosphorylated γH2AX-positive cells was observed in HSC^−/−^ (28.4 ± 6.9%) than in HSC^+/+^ mice (9.9 ± 3.4%, *p* = 0.014 by a two-tailed *t*-test).

Previously, mRNA profiling in HSCs isolated from *Cygb*^−/−^ mice showed important features of priming conditions with elevated expression of interleukin (Il)-6, tumour necrosis factor α (Tnfα), Il-1β, C-X-C motif chemokine ligand (Cxcl)-1, Cxcl-2, Cxcl-7, C-C motif chemokine ligand (Ccl)-2, Ccl-3, and Ccl-4 mRNA levels compared with those from WT^20^. These conditions were probably similar to the SASP of cells in which proinflammatory factors—such as ILs, chemokines and other inflammatory mediators, *e.g.*, matrix metalloproteinases (Mmps) and NO—are the major secreted components^28^. Moreover, the expression of these cytokines and chemokines was increased in the liver and liver tumours of aged *Cygb*^−/−^ mice compared with WT liver (Fig. 4E). These data suggest that loss of Cygb induced HSC senescence and SASP formation, thus affecting the tissue microenvironment for the promotion of tumour growth.

### Increased expression of chemokines in cocultures of hepatocytes and Cygb-deficient HSCs

**Figure 5:**
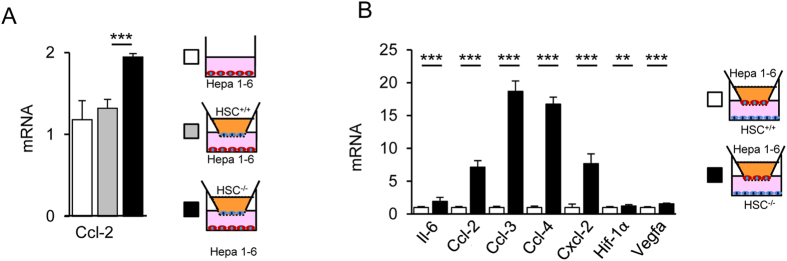
Increased expression of chemokines in cocultures of hepatocytes and Cygb-deficient HSCs. (**A**) Ccl-2 mRNA expression of mouse hepatoma Hepa 1–6 cells in single culture (open bar), coculture with HSC^+/+^ (grey bar), or coculture with HSC^−/−^ (closed bar) for 48 h. (**B**) HSC^+/+^ (open bar) and HSC^−/−^ cells (closed bar) were cocultured with Hepa 1–6 cells for 48 h. In HSC^−/−^ cocultured with Hepa 1–6 cells, increases were observed in the mRNA transcript levels of genes regulating inflammatory (Il-6, Ccl-2, Ccl-3, Ccl-4, Cxcl-2), hypoxia (Hif-1α), and angiogenesis (Vegfa), which were measured by qRT-PCR. Data represent mean ± SD; n = 4–9 for each group; *p < 0.05; **p < 0.01; ***p < 0.001.

The senescent phenotype of HSCs is believed to be involved in HCC development and propagation in mouse liver^27^. Therefore, we examined the interaction between hepatoma cells and HSCs in the presence or absence of Cygb. Accordingly, we cocultured mouse hepatoma Hepa 1–6 cells with HSCs^+/+^ or HSCs^*−/−*^ cells using a transwell insert. The Ccl-2 mRNA level in Hepa 1–6 cells was increased two-fold when cocultured for 48 h with HSCs^−/−^ cells compared with HSCs^+/+^ (Fig. 5A). The other genes tested showed no significant changes in expression (data not shown). In contrast, when HSCs were cocultured with Hepa 1–6 cells for 48 h, the gene expression profile of HSCs^−/−^ showed upregulation of a variety of cytokine and chemokine mRNAs, including Il-6, Ccl-2, Ccl-3, Ccl-4, Cxcl-2, and vascular endothelial cell growth factor α (Vegfα), compared with those of HSCs^+/+^ (Fig. 5B). These results implied that the soluble products excreted from Hepa 1–6 cells stimulated the senescent HSCs^−/−^ to produce more soluble signalling factors.

### Inhibition of NO synthesis reversed the phenotype of Cygb^−/−^ mice

**Figure 6:**
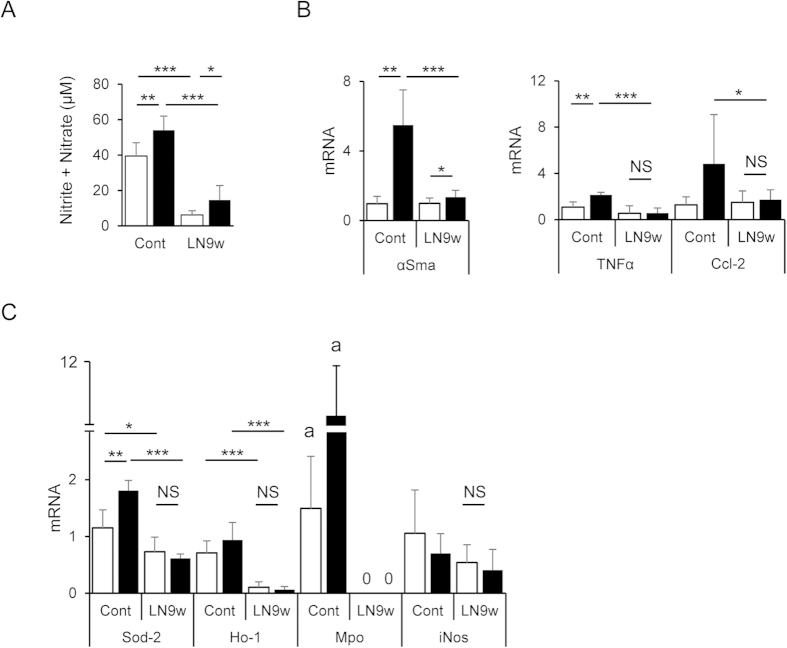
Inhibition of nitric oxide synthesis balancing the mRNA transcript levels of genes regulating inflammation, and oxidative stress condition. Inhibition of NO synthesis by treatment with a low dose, 0.01 mg/mL, of L-NAME for 9 weeks (LN9w) in drinking water was performed in WT (open bar) and KO (closed bar) mice starting from 12 weeks of age. Untreated mice were used as control (cont). (**A**) L-NAME lowers the total nitrite and nitrate level in the serum of L-NAME treated WT and KO mice compared with untreated control. (**B**) Effect of L-NAME in the expression of αSma, Tnf-α, and Ccl-2 mRNA in the liver. (**C**) Effect of L-NAME in the expression of oxidative stress regulated genes in WT and KO mouse livers. Note the undetectable level of the Mpo transcript gene (depict as 0) together with the very low expression of Ho-1 in both WT and KO mice under L-NAME treatment. Data represent the mean ± SD; n = 5–10 for each group; *p < 0.05; **p < 0.01; ***p < 0.001; a, p < 0.05 for comparisons of the WT and KO control with the L-NAME treatment using one sample t-test with a hypothetical mean value of 0.

To evaluate the potential of NO depletion to reverse the phenotype observed in *Cygb*^−/−^ mice, we examined young (12-week-old) mice exposed to 9 weeks of 0.01 mg/mL L-NAME in their drinking water. The NO level (total nitrite and nitrate) in serum was decreased significantly in both WT (6-fold compared with the untreated control) and *Cygb*^−/−^ mice (4-fold) following L-NAME treatment (Fig. 6A). Subsequently, all the changes in young *Cygb*^−/−^ mice, i.e., elevated expression of αSma, Tnfα, and Ccl-2, were decreased to the same level as that of WT following L-NAME treatment (Fig. 6B). The liver of young age *Cygb*^−/−^ mice still showed elevated expression of antioxidant genes, such as Sod-2 and Cat-1, as shown in Fig. 3E; the expression decreased in the aged mice (Fig. 3E). Here, after L-NAME treatment, the expression levels of both antioxidant and oxidative stress-related genes were reduced to the WT level (Fig. 6C). Interestingly, we observed an undetectable level of Mpo transcript gene (depicted as 0) together with very low expression of Ho-1 in both WT and KO mice under L-NAME treatment (Fig. 6C). Thus, L-NAME treatment *in vivo* can reverse the phenotype observed in *Cygb*^−/−^ mice at least at the young age.

## Discussion

### Organ abnormalities induced by Cygb deficiency

This study demonstrated that the absence of Cygb augments oxidative stress, induces DNA damage and cellular senescence, and triggers HSC activation, implying the importance of Cygb in pericytes for the homeostasis of individual organs and tissue microenvironment. In particular, this study suggested a role for Cygb in NO metabolism, as indicated by the increased concentration of NO metabolites (nitrite and nitrate) in both the serum and urine in *Cygb*^−/−^ mice compared with WT (Fig. 3). Importantly, all the phenotype observed in young *Cygb*^−/−^ mice including the increased expression of fibrosis, inflammation and oxidative stress regulated genes was reversed when the mice were treated with NO inhibitor, L-NAME (Fig. 6). NO is an important gas radical that causes dilation of blood vessels in the body and acts as a cytoprotective agent under physiological conditions. However, in pathological conditions, such as inflammation, NO binds to O_2_^−^ to form peroxynitrite and nitrotyrosine, which, in turn, cause DNA nucleotide modifications and induce the dysfunction and degradation of many functional proteins in the body^29^. Persistent nitration of proteins and DNA is potentially carcinogenic; significantly higher levels of nitrated proteins and/or iNos expression have been reported in lung^30^, breast^31^, head and neck^32^ and ovarian^33^ cancers in human, as well as in liver and lung cancers^19^,^20^ in mice. Thus, tumour development via Cygb deficiency involving deregulation of NO metabolism potentially represents a novel target in cancer research.

Young *Cygb*^−/−^ mice frequently exhibited cyst formation, especially cystic kidney diseases, which involve a dilation of tubules^34^ and renal myomatous lesions, as shown in Fig. 1Ck,l. NO controls nephron transport at the proximal tubule and thick ascending limb, and it affects both the stimulation and inhibition of net fluid and bicarbonate^35^. Studies in patients^36^ and in rats^37^ suggested a role for NO in the pathogenesis of hypertension and cyst development in autosomal dominant polycystic kidney disease (ADPKD).

We found two mice that showed severe loss of balance, and they did not survive past 8 months. These mice first exhibited distinct head and body tilt and then ran in circles until becoming moribund. This phenomenon was reported in head slant mice, which contained inactivated NADPH oxidase organiser 1 (*Noxo1*) due to a deoxyadenosine insertion in exon 1 of *Noxo1*, resulting in the truncation of the 349-amino acid Noxo1 protein to a peptide of 34 amino acids^38^. Interestingly, such downregulated expression of *Noxo1* mRNA was also found in 1-month-old *Cygb*^−/−^ mice (Table 2).

Although a number of events at the molecular, cellular, and physiologic levels were changed in young Cygb-deficient mice, the most severe abnormalities were found in the aged mice. It is well documented that the incidence of malignant tumours increases progressively with age, in both animals and humans^39^,^40^. Three major hypotheses have been proposed to explain the association between cancer and age, including (1) the duration of carcinogenesis^41^; (2) age-related progressive changes in the internal milieu of the organism, including proliferative senescence^42^,^43^; and (3) the combined effects of a cumulative mutational load, increased epigenetic gene silencing, telomere dysfunction, and altered stromal milieu^44^. In this study, the age-related presence of tumours in multiple organs and fibrosis in *Cygb*^−/−^ mice might be related to the second hypothesis because the primary HSCs from *Cygb*^−/−^ mice exhibited the senescent phenotype.

### Heart hypertrophy

The most dominant phenotype found in aged *Cygb*^−/−^ mice was heart hypertrophy, as demonstrated by the increased size of cardiomyocytes and the HW/BW ratio. These results may be caused by the chronically slower metabolism of NO and the higher NO concentration in the aorta and vascular system, leading to an increased circulating volume in *Cygb*^−/−^ mice^45^. In general, cardiac hypertrophy occurs in response to long-term increases in haemodynamic load related to a variety of physiological and pathological conditions. The process of cardiac hypertrophy is characterised by structural changes in the cardiomyocytes that are translated into alterations in chamber size and geometry, collectively called remodelling. NO has emerged as an important regulator of cardiac hypertrophy, apoptosis and remodelling^46^. The diverse cardiac effects of NO depend on its source and concentration and on the local scavenging activity of radicals, including NO^47^. Exogenous NO exerted inhibitory effects on cardiomyocyte hypertrophy *in vivo*^48^ and *in vitro*^49^, which differed from our results. However, Moreau *et al*. showed that NO might be a necessary factor for cardiac hypertrophy in a rat model in which the concomitant administration of L-NAME, an inhibitor of nitric oxide synthesis, together with angiotensin II prevented the vascular hypertrophy induced by treatment with angiotensin II only^50^. Moreover, Mungrue *et al*. showed that conditional overexpression of iNos on cardiomyocytes was associated with peroxynitrite generation, cardiac fibrosis, myocyte death, increased cardiac mass and, ultimately, cardiac dilatation^51^. These data suggested that increased myocardial iNos activity initiates a process of cardiac remodelling that is characterised by ventricular dilatation, hypertrophy, and sudden cardiac death^51^. Consistent with our study, Mungrue *et al*. showed that upregulation of iNos led to increased formation of 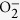 and ONOO^-^ in the heart^51^. ROS have been recognised as prohypertrophic signalling intermediates in cardiomyocytes^52^,^53^. Therefore, Cygb might be a key molecule in metabolizing NO and balancing the antioxidant defence in the heart.

### Fibrosis of the liver and other organs

HSCs play a critical role in extracellular matrix remodelling and fibrosis progression in chronic liver diseases^54^. Reactive oxygen intermediates, apoptotic bodies from hepatocytes, and paracrine stimuli from Kupffer cells trigger HSC activation^55^. Cygb was initially found in activated HSCs with increased expression^1^; thus, it was hypothesised that Cygb expression might protect HSCs from exposure to endogenous and exogenous ROS during liver injury. The ROS scavenger function of Cygb is evidenced by its ability to detoxify radicals via reaction with its haem^56^. Xu *et al*. demonstrated that forced overexpression of Cygb significantly increased the total oxy-radical scavenging capacity compared with that for the expression of control eGFP^17^ and that overexpression of Cygb protected primary rat HSCs against oxidative stress, as indicated by reduced production of MDA and 4-hydroxy-2-nonenal (4-HNE), biomarkers of lipid peroxidation. Furthermore, Cygb overexpression reduced tissue fibrosis in both toxic and cholestatic models of liver injury^17^.

Previously, we found that loss of *Cygb* was associated with the priming of HSCs, which amplified the expression of fibrogenesis-related genes, cytokines and a variety of chemokines^20^. The priming of HSCs probably contributes to the chronic progression of fibrosis in the *Cygb*^−/−^ mice in this study. Moreover, when *Cygb*^−/−−/−^ mice were administered a high-fat diet, such as the choline-deficient L-amino acid defined diet (CDAA), to induce steatohepatitis, they rapidly developed serious liver inflammation and fibrosis at an early time point^20^. Similarly, as patients with NASH developed more fibrosis, Cygb expression decreased in HSCs^20^. A study in human liver tissues damaged by hepatitis C virus (HCV) infection at various fibrosis stages revealed that the number of Cygb-positive cells decreased with fibrosis progression^5^.

Interestingly, a number of *Cygb*^−/−^ mice developed fibrosis in the kidney (Fig. 1Ck,l). Renal fibrosis, characterised by glomerulosclerosis and tubulointerstitial fibrosis, is the final common manifestation of a wide variety of chronic kidney diseases, similar to the wound-healing response in chronic liver injuries^57^. In line with our results, decreased kidney fibrosis induced by subtotal nephrectomy (remnant kidney) was demonstrated using transgenic rats overexpressing Cygb^58^. Therefore, Cygb supplementation might serve as a potential therapy for suppressing fibrosis in various organs.

### Cellular senescence and cancer development

Stromal fibroblasts from humans and mice have been studied extensively with respect to cellular senescence. Upon senescence, such cells show striking changes in gene expression^59^, some of which relate to the growth arrest and senescent morphology. Senescent fibroblasts secrete growth factors, cytokines, extracellular matrix and Mmps, all of which can alter tissue microenvironments and affect the function of nearby epithelial cells^60^,^61^. Recently, it was reported that obesity-associated HCC development is promoted by senescent HSCs^27^. Here we found that in the absence of Cygb, HSCs exhibited a senescent-like phenotype with expression of p16, p21, and p27 and production of SASP, which might contribute to liver tumour formation. Such senescence of mesenchymal fibroblast-like cells could occur in virtually all organs because tumour formation occurred in the lung, liver, intestine, and lymphoid system. *Cygb* might be an interesting tumour suppressor gene candidate not only in the liver but also in other organs due to its control of the senescence of pericytes, such as HSCs. Numerous investigations on the tumour-suppressing activity of Cygb have been reported since 2005; the studies showed that most cancer cells and tissues have reduced expression of Cygb and/or loss of heterozygosity, in addition to promoter hypermethylation both *in vitro* and *in vivo*^14^,^18^,^19^,^62^.

In conclusion, the absence of Cygb in pericytes provokes organ abnormalities and tumour formation due to possible derangement of ROS, including the NO and antioxidant system. Induced cellular senescence might be involved in the initiation of local inflammatory reactions and microenvironment modifications, leading to organ fibrosis. Further studies using targeted *Cygb* overexpression and knockdown in *in vivo* and cultured pericytes are needed to further clarify the molecular function of Cygb.

## Materials and Methods

### Animal and histopathological analysis

C57BL/6 *Cygb* knockout (*Cygb*^−/−^) mice were generated in our laboratory as described previously^19^. *Cygb* heterozygous mice were backcrossed to the C57BL/6J background for more than nine generations. To assess the role of *Cygb* in development, we intercrossed *Cygb* heterozygous mice. The homozygotes appeared normal morphologically and histopathologically at 1 M. Mice were genotyped for the absence of Cygb at the DNA, RNA, and protein levels as described previously^19^. Both males and females were kept for observation and sacrificed at the designated age: 1 to 6 months old (1–6 M group), 7 to 12 months old (7–12 M group), 13 to 18 months old (13–18 M group), and 19 to 24 months old (19–24 M group). Each group of males or females contained 15 to 56 WT or *Cygb*^−/−^ mice. The BW, LW, and HW were recorded. Heart sizes, including length and width, were measured. A complete necropsy was performed in each mouse. The blood was collected for the assays. All tissues were harvested, and abnormalities were noted. Tissue portions were kept at −80 °C for further analysis, while other portions were fixed in neutral buffered formalin, embedded in paraffin, sectioned and stained with H&E. Mouse neoplastic and non-neoplastic lesions were diagnosed according to standard criteria^63^,^64^, and all abnormal histologic changes were recorded using a computerised autopsy data system. Records of morbidity or mortality were assigned subjectively to each mouse that died or to those that were sacrificed while moribund after review of the autopsy and histopathologic findings^65^. Selected tissues from mice with lymphoma were subjected to immunohistochemistry using CD3 for T cells or CD22 for B cells (Supplementary Table 1).

A subgroup of WT and *Cygb*^−/−^ mice at 12 weeks of age, n = 10 each group, received L-NAME (Sigma-Aldrich, Tokyo, Japan) treatment at the dose of 0.01 mg/mL in drinking water. Untreated mice were used as control. After 9 weeks of L-NAME treatment, the mice were sacrificed for further analysis. All mice were housed in a facility with a 12-h light/dark cycle and allowed free access to food and water. All protocols and experimental procedures were approved by the Institutional Animal Care and Use Committee of Osaka City University and performed in accordance with the guidelines of the National Institutes of Health for the use of animals in research.

### Immunohistochemistry and immunofluorescence analysis

H&E staining, immunohistochemistry and immunofluorescence analysis were performed as described previously^19^. The primary antibodies used are listed in Supplementary Table 1. Polyclonal antibodies against Cygb were generated in our laboratory^1^,^5^,^19^. To compare the size of the heart, images representing the whole vertical section were acquired at 100× magnification and digitalised. These separately captured and digitalised images were consolidated to create one large image using the e-Tiling system (Mitani Corporation, Tokyo, Japan). To quantitate liver fibrosis, 5-μm sections were cut, stained with Picrosirius red (Sigma-Aldrich, Tokyo, Japan) and counterstained with Fast Green dye (Sigma-Aldrich). Sirius red-positive areas were quantified in whole liver lobes using a BZ-X700 microscope and its BZ-X Analyser analysis software (Keyence, Osaka, Japan). For quantification, phosphorylated γH2AX-positive cells in immunohistochemical/immunofluorescence stained mouse liver (n = 5 each group) and primary HSCs sections (n = 3 each group) were counted in at least 10 high-power fields (400×) per one section.

### Nitric oxide assay

The concentrations of total nitrate and nitrite in the serum and urine were measured by colourimetric methods using a Nitric Oxide Assay Kit (Abcam, Cambridge, UK) according to the manufacturer’s protocol. Briefly, two-step process was performed, in which first step converted nitrate to nitrite utilizing nitrate reductase. The second step used Griess reagents to convert nitrite to a deep purple azo compound recorded at 540 nm. The amount of the azochromophore accurately reflects nitric oxide amount in samples.

Urine from individual mice was collected over 24 h using silicon wafers that covered the bottom of the mouse cage. Collections occurred at 3 h intervals, including 6:00–9:00, 9:00–12:00, 12:00–15:00, 15:00–18:00, 18:00–21:00, and one 9-h interval from 21:00 until the next day at 6:00 AM. A fresh silicon wafer was placed at each collection time. The total urine volume in a 24 h period was measured and used to calculate the total nmol of nitrate + nitrite in urine per day.

### Glutathione assay

GSH, the major endogenous antioxidant produced by the cells, participates directly in the neutralization of free radicals and reactive oxygen compounds. GSH was measured in the serum (reduced GSH) and homogenised liver lysates. Both the reduced GSH and oxidised glutathione (GSSG) were determined using a glutathione assay kit (Cayman, Ann Arbor, MI) according to the manufacturer’s protocol. Briefly, a carefully optimized enzymatic recycling method using glutathione reductase was utilized. The sulfhydryl group of GSH reacts with 5,5′ –dithio-bis-2-nitrobenzoic acid (DNTB) and produces a yellow coloured 5-thio-2-nitrobenzoic acid (TNB) which was recorded at 405 nm. The oxidized glutathione (GSSG) was measured after its reduction by glutathione reductase.

### Malondialdehyde assay

To assess the oxidative stress status in *Cygb*^−/−^ mice, lipid peroxidation in the serum and homogenised liver lysates was quantified by measuring its end product, MDA (BioVision, CA, USA) according to the manufacturer’s protocol. Briefly, MDA in the sample is reacted with Thiobarbituric acid (TBA) to generate the MDA-TBA adduct which was quantified colorimetrically at 530 nm.

### ALT and AST measurement

ALT and AST activities were measured in serum using a commercially available kit (Wako, Osaka, Japan) according to the manufacturer’s protocol.

### Quantitative real-time PCR

Total RNA was extracted from cells and tissues using a miRNeasy Mini Kit (Qiagen, Valencia, CA). cDNAs were synthesised using total RNA, a ReverTra Ace qPCR RT Kit (Toyobo, Osaka, Japan) and oligo(dT)_12–18_ primers according to the manufacturer’s instructions. Gene expression was measured by real-time PCR using the cDNAs, SYBR qPCR Mix reagents (Toyobo) and gene-specific oligonucleotide primers (Supplementary Table 2) with an ABI Prism 7500 Fast Real-Time PCR System (Applied Biosystems, Foster, CA). The *Gapdh* level was used to normalise the relative abundance of mRNAs.

### Gene expression profile for a specific pathway

An RT^2^ *Profiler*™ PCR Array for the Mouse Oxidative Stress and Antioxidant Defense (SA Biosciences, Frederick, Maryland; cat # PAMM-065) was used to examine the expression of 84 genes related to oxidative stress according to the manufacturer’s protocol. Briefly, 1 μg of total RNA from 1- and 14-month-old WT or *Cygb*^−/−^ mice was used to make first strand complementary DNA (cDNA) using the RT^2^ First Strand Kit (SA Biosciences). The PCR mixture containing cDNA, distilled water, and SYBR Green master mix (SA Biosciences) was loaded onto each well of 96-well plates containing the pre-dispensed gene-specific primer sets. PCR was performed with an ABI Prism 7500 Fast Real-Time PCR System (Applied Biosystems). PCR was performed in 96-well plates with 84 genes related to oxidative stress. Five housekeeping genes (*actin B*, *Gapdh*, *Hsp90ab1*, *Hprt1*, and *Gusb*) were used for normalizing the PCR array data, one negative control was used to verify genomic DNA contamination, and three wells of reverse transcription controls (RTC) were employed to verify the efficiency of the RT reaction. Excel-based PCR array data analysis (SA Biosciences) was used to calculate the threshold cycle (Ct) values for all genes in the array. Then, fold-changes in gene expression for pairwise comparisons using the ΔΔCt method were used to determine the relative expression levels of genes of interest for each sample.

### Immunoblot analysis

Protein samples (10 to 40 μg) were subjected to SDS-PAGE and transferred to Immobilon P membranes (Millipore Corp, Bedford, MA). After blocking, membranes were probed with primary antibodies against αSMA (1:1000; Dako), nitrotyrosine (NT, 1:1000; Abcam), or GAPDH (1:2000; Santa Cruz Biotechnology, Santa Cruz, CA). Membranes were then incubated with horseradish peroxidase-conjugated secondary antibodies at 1: 2000 dilutions. Immunoreactive bands were visualised using the ECL detecting reagent (GE Healthcare UK Ltd, Buckinghamshire, UK) and documented with a Fujifilm Image Reader LAS-3000 (Fujifilm, Tokyo, Japan) coupled to image analysis software (Multi Gauge, Fujifilm).

### Cells

HSCs were isolated from WT and *Cygb*^−/−^ mice using the pronase-collagenase digestion method as described previously^66^ and were cultured on uncoated-plastic dishes (BD Falcon, Franklin Lake, NY, USA) or glass chamber slides (Thermo Fisher Scientific, Waltham, MA, USA) in DMEM (Sigma-Aldrich) supplemented with 10% FBS (Invitrogen, Carlsbad, CA, USA) and antibiotics (100 U/ml penicillin and 100 μg/ml streptomycin) at 37 °C in a 5% CO_2_/95% room air. Mouse hepatoma Hepa 1–6 cells (CRL-1830) obtained from American Type Culture Collection (Manassas, VA, USA) were maintained on uncoated-plastic culture plates (BD Falcon) in DMEM supplemented with 10% FBS and antibiotic.

### Coculture experiments

Mouse hepatoma Hepa 1–6 cells, or HSCs isolated from *Cygb*^+/+^ (HSC^+/+^) and *Cygb*^−/−^ mice (HSC^−/−^) were cultured alone or cocultured in serum-free William’s E medium (Invitrogen) using 6-well plates and transwell inserts with 1-μm pore size, which allowed diffusion of medium components but prevented cell migration (BD Biosciences). Co-cultured of Hepa 1–6 cells with HSC^+/+^ or HSC^−/−^ cells were prepared as follows: Hepa 1–6 cells were plated on the bottom of the six-well transwell cell culture system in serum-free William’s E medium and cultured at 37 °C in a 5% CO_2_/95% room air. HSC^+/+^ or HSC^−/−^ cells were cultured onto the membrane of transwell cell culture inserts. All cells were allowed to grow overnight using the above mentioned condition. The next day the all cells were washed with the serum-free media and membrane transwell inserts containing HSC^+/+^ or HSC^−/−^ cells were placed into the six-well plates cultured containing the Hepa 1–6 cells to initiate the co-cultured experiment. Similar pattern was used for HSC^+/+^ or HSC^−/−^ cells co-cultured with Hepa 1–6 cells. After 48 h, the cells were harvested for RNA isolation.

### Statistical analysis

All data are expressed as the mean ± standard error of the mean. Two groups were compared using an unpaired Student *t*-test (two-tailed). *P* values less than 0.05 were considered statistically significant.

## Additional Information

**How to cite this article**: Thuy, Le. T. T. *et al*. Absence of cytoglobin promotes multiple organ abnormalities in aged mice. *Sci. Rep*. **6**, 24990; doi: 10.1038/srep24990 (2016).

## Supplementary Material

Supplementary Information

## Figures and Tables

**Table 1:** Frequency of abnormalities in Cygb-deficient mice aged 1 to 2 years.

Age	13–18 months		19–24 months	
Strain	WT	*Cygb*^−/−^	WT	*Cygb*^−/−^
Total # of mice	35	31	33	84
Heart hypertrophy (n)	0	8	2	34
%	0	25.8^b^	6	40.5^c^
Lymphoma (n)	0	7	0	24
%	0	22.6^b^	0	28.5^c^
Liver tumours (n)	0	2	1	19
%	0	6.5	3	22.6^a^
Lung tumours (n)	0	3	1	7
%	0	9.7	3	8.3
Cysts (n)	0	4	0	1
%	0	12.9^a^	0	1.2

^a^*p* < 0.05; ^b^*p* < 0.01; ^c^*p* < 0.001.

**Table 2:** Imbalance of oxidative stress and antioxidant genes in *Cygb* ^−/−^ mice compared with WT controls.

Gene name	Fold-regulation in KO/WT		
	Gene symbol	1 month	14 months
Aquarius	Aqr	−1.021	−2.2038
Ataxia telangiectasia and rad3 related	Atr	−1.1251	−3.5308
Copper chaperone for superoxide dismutase	Ccs	1.1975	−2.7321
EH-domain containing 2	Ehd2	−2.0849	1.1096
Eosinophil peroxidase	Epx	1.0644	3.249
Excision repair cross-complementing rodent repair deficiency, complementation group 2	Ercc2	−1.2483	−2.0139
Excision repair cross-complementing rodent repair deficiency, complementation group 6	Ercc6	1.257	−2.5491
Flavin containing monooxygenase 2	Fmo2	1.454	−3.1602
Glutathione peroxidase 3	Gpx3	−2.6759	1.2311
Intraflagellar transport 172 homolog (*Chlamydomonas*)	Ift172	−1.5692	−2.2191
Kinesin family member 9	Kif9	−1.7532	−2.6574
Myeloperoxidase	Mpo	−1.2058	122.7858
Nitric oxide synthase 2, inducible	Nos2	−4.9588	3.9449
NADPH oxidase organiser 1	Noxo1	−2.8879	−2.7702
Nudix (nucleoside diphosphate linked moiety X)-type motif 15	Nudt15	1.2226	−2.0562
Nucleoredoxin	Nxn	1.0644	4.6913
Peroxiredoxin 6, pseudogene 1	Prdx6-ps1	−1.7053	−2.0139
RecQ protein-like 4	Recql4	−2.6208	−1.9862
Serine (or cysteine) peptidase inhibitor, clade B, member 1b	Serpinb1b	−4.8232	−20.8215
Solute carrier family 38, member 1	Slc38a1	−1.3379	−2.4967
Sulfiredoxin 1 homolog (*S. cerevisiae*)	Srxn1	−2.7321	−1.7291
Superoxide dismutase 2, mitochondrial	Sod2	−1.6133	−2.9897
Superoxide dismutase 3, extracellular	Sod3	−1.5476	1.0281
Thioredoxin interacting protein	Txnip	1.5911	−2.1735
