# Supplementary material for: Absence of cytoglobin promotes multiple organ abnormalities in aged mice
Source: Sci Rep. 2016 May 5;6:24990. doi: 10.1038/srep24990 (PMC4857093; doi:10.1038/srep24990)
Supplement: Supplementary Information [file srep24990-s1.pdf]

Absence of cytoglobin promotes multiple organ abnormalities in aged mice

Le Thi Thanh Thuy[1], Tuong Thi Van Thuy[1], Yoshinari Matsumoto [2], Hoang Hai[1], Yoshihiro Ikura[3], Katsutoshi Yoshizato[1,4], and Norifumi Kawada[1,*]

[1]Department of Hepatology, Graduate School of Medicine, Osaka City University, Osaka, Japan, [2]Department of Medical Nutrition, Graduate School of Human Life Science, Osaka City University, Osaka, Japan, [3]Department of Pathology, Takatsuki General Hospital, Takatsuki, Osaka, Japan and [4]PhoenixBio Co. Ltd., Hiroshima, Japan.

[*]**Corresponding author:**

Norifumi Kawada, M.D., Ph.D.

Department of Hepatology, Graduate School of Medicine, Osaka City University, 1-4-3 Asahimachi, Abeno, Osaka 545-8585, Japan

Phone: +81-6-6645-3897; Fax: +81-6-6646-6072; E-mail: kawadanori@med.osaka-cu.ac.jp

Supplementary Figure S1

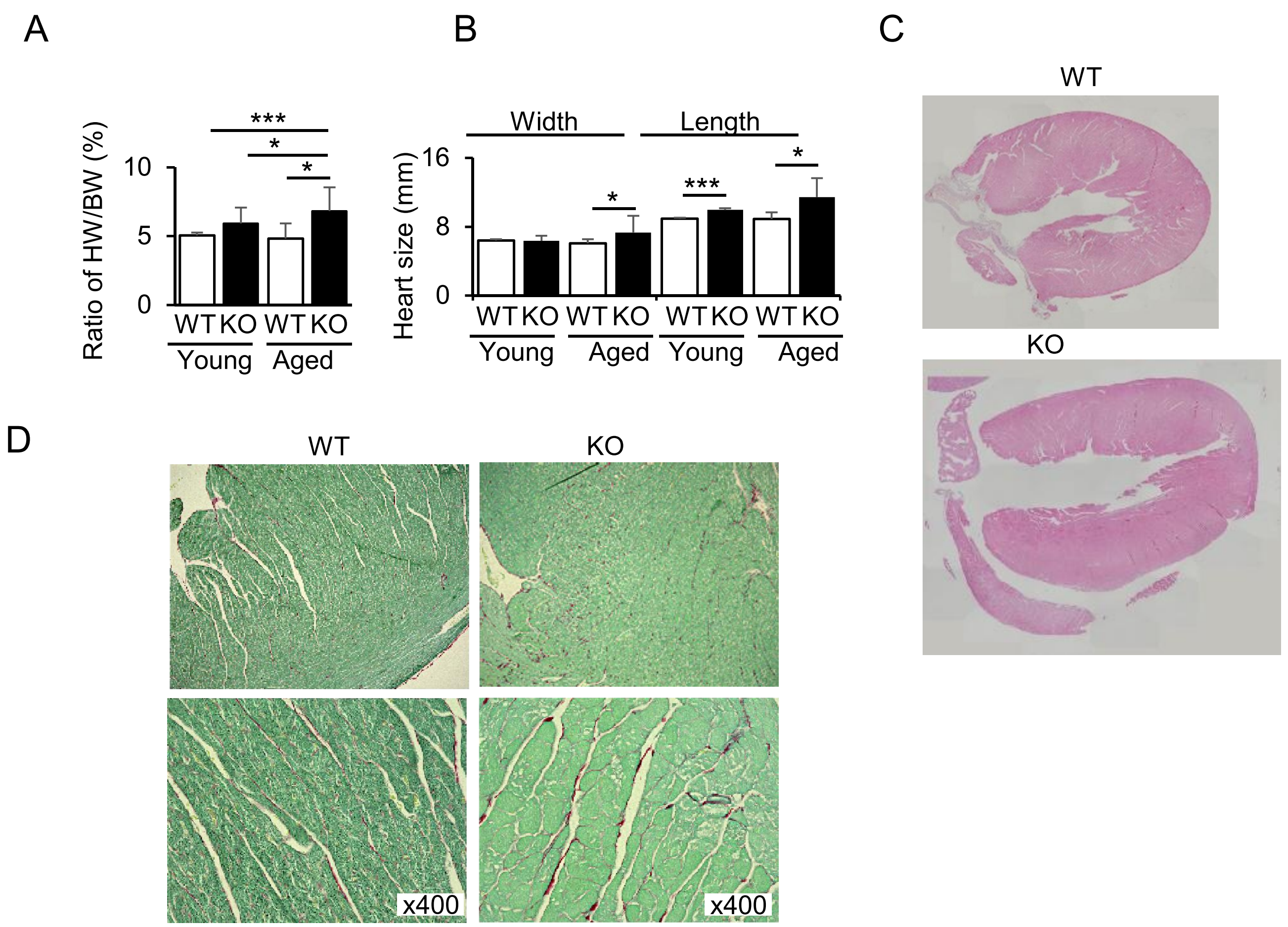

Supplementary Figure S1: A, Percentage of heart weight (HW) to body weight (BW) ratio (A), heart size (B), including the width and length of the mouse heart, in young and aged WT and KO mice. C, Actual size of the aged WT and KO hearts (see Methods for detailed description). (D) SiR-FG staining of heart sections from WT and KO mice.

Supplementary Figure S2

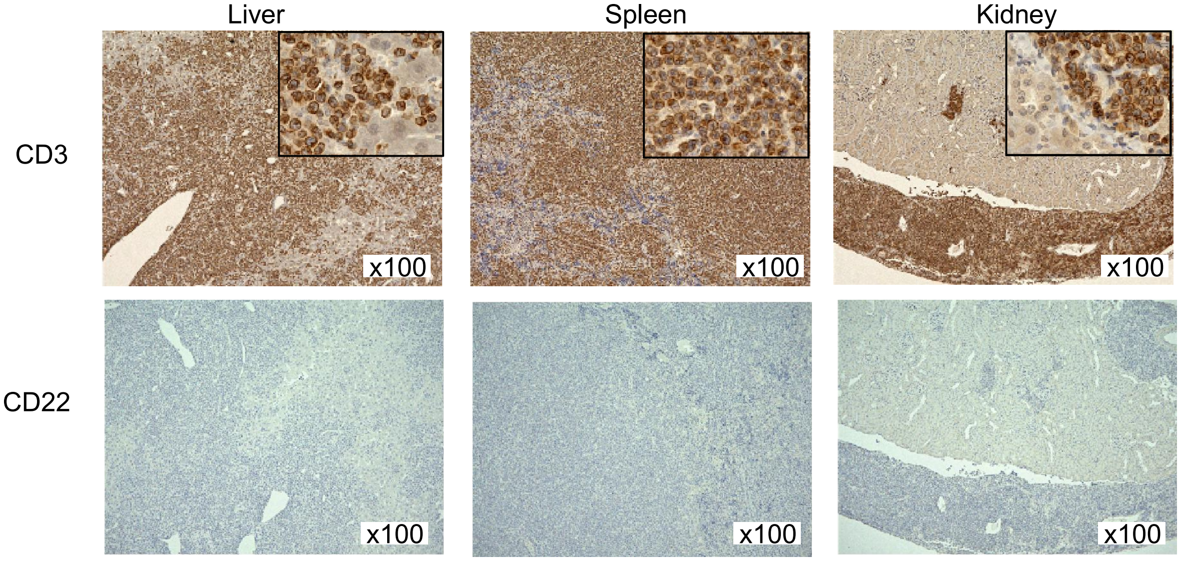

Supplementary Figure S2: Positive immunohistochemically staining for CD3, marker of T cells, but not CD22, marker of B cells, in the liver, spleen, and kidney of Cygb$^{-/-}$ mouse bearing lymphoma at 11 months old. Inset, x1200.

Supplementary Figure S3

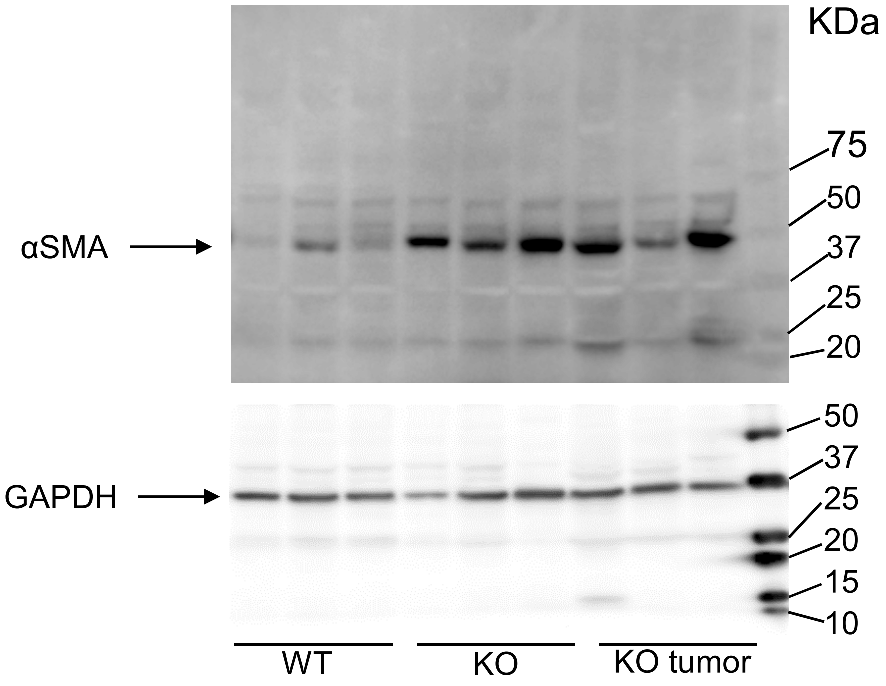

Supplementary Figure S3: Immunoblot analysis of α SMA expression from homogenate liver tissues of aged wild-type (WT), Cygb$^{-/-}$ (KO), and Cygb$^{-/-}$ liver tumors (KO tumor). GAPDH was used as loading control.

Supplementary Figure S4

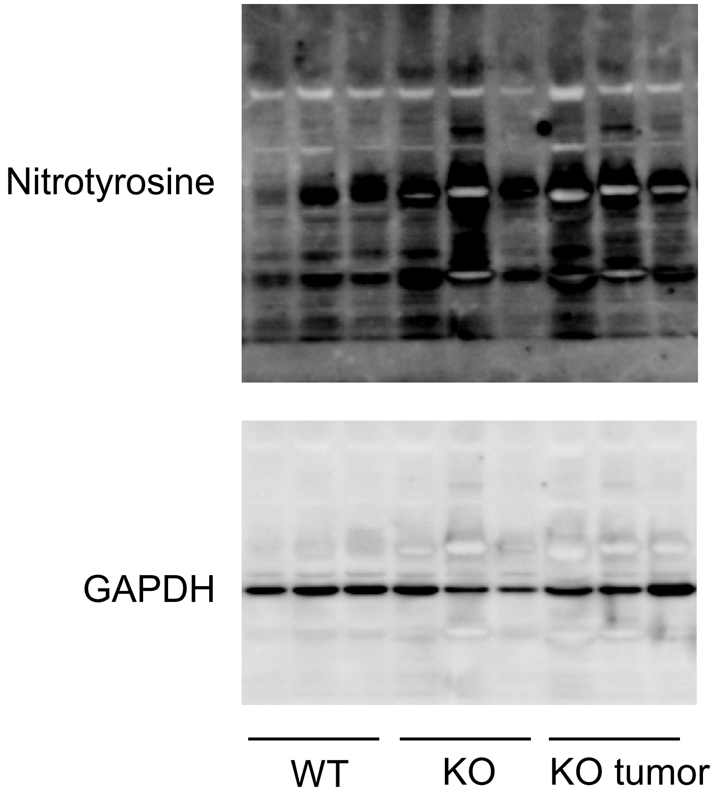

Supplementary Figure S4: Immunoblot analysis of nitrotyrosine protein adducts from homogenate liver tissues of aged wild-type (WT). Cygb$^{-/-}$ (KO), and Cygb$^{-/-}$ liver tumors (KO tumor). GAPDH was used as loading control.

Supplementary Table S1. Summary of first primary antibodies used for immunohistochemistry or immunofluorescences in this study

Antigen *	Source	Name/clone†; Catalog no.	Incubation ‡
CYGB	Our laboratory	Polyclonal (Rb) anti mouse	O/N 4°C, 1:300
CD3	AbD Serotec	Monoclonal (Rt); Clone: CD3-12	O/N 4°C, 1:100
CD22	Abcam	Polyclonal (Rb); ab65852	O/N 4°C, 1:100
HO-1	Assay designs	Polyclonal (Rb); #SPA-895	30 min RT 1:100
CRBP-1	Santa Cruz	Polyclonal (Rb); sc-30106	O/N 4°C, 1:100
Desmin	Sigma	Monoclonal (Mo); Clone: DE-U-10	O/N 4°C, 1:100
p16	Santa Cruz	Polyclonal (Rb); sc-1207	O/N 4°C, 1:300
p21	Abcam	Polyclonal (Rb); ab2961	O/N 4°C, 1:100
αSma	Sigma	Monoclonal (Mo); Clone: 1A4	O/N 4°C, 1:300
γH2AX	Novus Biologicals	Monoclonal (Rb); NB100-79967	O/N 4°C, 1:200
Netrophil	Abcam	Monoclonal (Rt); ab2557	O/N 4°C, 1:100

* All antigens were retrieved by autoclaving for 15 min in 0.01 M citrate buffer containing 0.05% Tween 20 (pH 6.0) except for Neutrophile in which Proteinase K 400 microgram/mL in TE buffer (pH 8.0) was used.

†Rb, rabbit; Mo, mouse; Rt, rat

‡ O/N, overnight; RT, room temperature

Supplementary Table S2. Mouse primers used for quantitative RT-PCR

Gene	Sequence
mCat-1	F: 5'-ATGGCTTTTGACCCAAGCAA-3'
	R: 5'-CGGCCCTGAAGCTTTTTGT-3'
mCcl-3	F: 5'- TGAAACCAGCAGCCTTTGCTC-3'
	R: 5'- AGGCATTCAGTTCCAGGTCAGTG-3'
mCcl-4	F: 5'- CCATGAAGCTCTGCGTGTCTG-3'
	R: 5'- GGCTTGGAGCAAAGACTGCTG-3'
mGro (Cxcl-1)	F: 5'-TGAGCTGCGCTGTCAGTGCCT-3'
	R: 5'-AGAAGCCAGCGTTCACCAGA-3'
mCxcl-2	F: 5'-GAGCTTGAGTGTGACGCCCCCAGG-3'
	R: 5'-GTTAGCCTTGCCTTTGTTCAGTATC-3'
mCxcl-9	F: 5'-ATTGTGTCTCAGAGATGGTGCTAATG -3'
	R: 5'-TGAAATCCCATGGTCTCGAAAG -3'
mHif1α	F: 5'-CAGTACAGGATGCTTGCCAAAA-3'
	R: 5'-ATACCACTTACAACATAATTCACACACACA-3'
mCygb	F: 5'-TGCATGACCCAGACAAGGTA-3'
	R: 5'-GGTCACGTGGCTGTAGATGA-3'
mGapdh	F: 5'-TGCACCACCAACTGCTTAG-3'
	R: 5'-GGATGCAGGGATGATGTTC-3'
mGpx-6	F: 5'-GCCCAGAAGTTGTGGGGTTC-3'
	R: 5'-TCCATACTCATAGACGGTGCC-3'
mHo-1	F: 5'-GGTGATGGCTTCCTTGTACC-3'
	R: 5'-AGTGAGGCCCATACCAGAAG-3'
mIl-1β	F: 5'-CCATGGCACATTCTGTTCAAA-3'
	R: 5'-GCCCATCAGAGGCAAGGA-3'
mIl-6	F: 5'-CCGCTATGAAGTTCCTCTCTGC-3'
	R: 5'-ATCCTCTGTGAAGTCTCCTCTCC-3'
m-iNos	F: 5'-CCTGGTACGGGCATTGCT-3'
	R: 5'-GCTCATGCGGCCTCCTTT-3'
mCcl-2	F: 5'-GAGAGCCAGACGGGAGGAAG-3'
	R: 5'-TGAATGAGTAGCAGCAGGTGAG-3'
mMpo	F: 5'-CCATGGTCCAGATCATCACA-3'
	R: 5'-GCCGGTACTGATTGTTCAGG-3'
m-p16	F: 5'-GGGTTTCGCCCAACGCCCCGA-3'
	R: 5'-TGCAGCACCACCAGCGTGTCC-3'
m-p21	F: 5'-GCAGATCCACAGCGATATCC-3'
	R: 5'-CAACTGCTCACTGTCCACGG-3'
m-p27	F: 5'-AAGGGCCAACAGAACAGAAG-3'
	R: 5'-GGATGTCCATTCAATGGAGTC-3'

mTnfα	F: 5'-CTCTTCTCATTCCTGCTTGTGG-3'
	R: 5'-AATCGGCTGACGGTGTGG-3'
m-αSma	F: 5′-TCCCTGGAGAAGAGCTACGAACT-3′
	R: 5′-AAGCGTTCGTTTCCAATGGT-3′
mVegfα	F: 5′-AACGATGAAGCCCTGGAGTG-3′
	R: 5′-TGAGAGGTCTGGTTCCCGA-3′
mSod-2	F: 5'-CACATTAACGCGCAGATCATG-3′
	R: 5′-CCAGAGCCTCGTGGTACTTCTC-3′
